# History of fecal transplantation; camel feces contains limited amounts of *Bacillus subtilis* spores and likely has no traditional role in the treatment of dysentery

**DOI:** 10.1371/journal.pone.0272607

**Published:** 2022-08-10

**Authors:** Nienke Koopman, Pim van Leeuwen, Stanley Brul, Jurgen Seppen

**Affiliations:** 1 Swammerdam Institute for Life Sciences (SILS)—University of Amsterdam, Amsterdam, The Netherlands; 2 Tytgat Institute for Liver and Intestinal Research, Amsterdam University Medical Centers, Amsterdam, The Netherlands; University of Arkansas Fayetteville, UNITED STATES

## Abstract

**Introduction:**

A widely cited story on the origins of fecal transplantation suggests that German soldiers in North Africa used camel feces containing *Bacillus subtilis* to treat dysentery in World War 2. We investigated if this story is accurate and if there is sufficient *Bacillus subtilis* in camel feces to be potentially therapeutic.

**Methods and results:**

A literature analysis shows that all references to the story are based on a single review paper that mentions the use of camel feces in passing and only provides indirect evidence for this claim. An extensive literature search failed to find independent evidence that camel feces has traditionally been used in the treatment of dysentery in North Africa. With 16S sequence analysis we did not detect *Bacillus subtilis* in feces from two different Egyptian camels. Using a more sensitive culture-based assay we could detect low amounts of *Bacillus subtilis* spores in these fecal samples, with comparable concentrations to those present in human feces and soil.

**Conclusions:**

Because we could not find evidence for the use of camel feces in the treatment of diarrhea and because we show that only low amounts of *Bacillus subtilis* spores are present in camel feces, we conclude that the use of camel feces should no longer be mentioned in the context of origins of fecal transplantation.

## Introduction

Because the composition of the intestinal microbiome has been linked to many disorders, therapeutic interventions to change this composition are widely studied. One of the promising approaches to therapeutically change the intestinal microbiome is transplantation of “healthy” feces [1, 2]. This fecal transplantation is used in many centers to successfully treat *Clostridioides difficile* infection [3, 4], although some concerns remain [5]. In addition to the treatment of *Clostrioides difficile*, the use of fecal transplantation is studied for many other disorders [6]. Fecal transplantation is not an entirely new approach, the first hints at its efficacy for *Clostridioides difficile* infection go back to 1958 [7]. Perhaps because of these roots, many papers on fecal transplantation provide short historical overviews where earlier efforts in the field of fecal transplantation are summarized.

One story that is presented in various forms, cited in many papers (see S1 Appendix for a selection), and internet sites can be paraphrased as follows: In the Second World War, German soldiers from the Africa corps noticed that Bedouins from North Africa consumed fresh camel feces to treat dysentery. The German army is reported to have recognized the presence of *Bacillus subtilis* in the feces and subsequently utilize this knowledge to obtain a treatment for soldiers suffering from dysentery. We explore the validity of this story by a literature review and microbial analysis of camel feces.

## Results and discussion

The historical use of camel feces containing *Bacillus subtilis* for the treatment of dysentery seems plausible because probiotic preparations containing *Bacillus subtilis* strains have been used in humans [8–10]. However, the efficacy of *Bacillus subtilis* as a probiotic to treat diarrhea is difficult to ascertain, proper studies are scarce. A randomized controlled trial where subjects with loose stools received a daily dose of 2,2.10^9^ spores or placebo showed that *Bacillus subtilis* spores did not reduce stool water content but reduced Bristol stool score [11].

The reference for the story that camel feces has therapeutic utility due to the presence of *Bacillus subtilis*, appears to be a paper by Ralph A. Lewin titled “More on Merde (2001)” [12]. All papers that mention the “camel feces” story refer to this paper, either by direct or indirect citation. It should be noted that this paper only briefly mentions the “camel feces” story;”*However*, *consumption of fresh*, *warm camel feces has been recommended by Bedouins as a remedy for bacterial dysentery; its efficacy (probably attributable to the antibiotic subtilisin from Bacillus subtilis) was confirmed by German soldiers in Africa during World War II”* [12].

The source of the “camel feces” story mentioned in the paper by Ralph A. Lewin is a German language internet article about *Bacillus subtilis* written by Dr. Jörg Bernhardt from the Ernst Moritz Arndt University Greifswald (*Bacillus subtilis* Beschreibung und Charakterisierung) (http://microbio1.biologie.uni-greifswald.de:8080/institute/85, archived by wayback machine 9 September 2002). The original piece (S2 Appendix) was translated as follows: *“The first documented medical application of B*. *subtilis took place in 1941 during a campaign in Libya by the African corps ambulance troops of the German armed forces*. *A dysentery epidemic to which numerous soldiers fell victim to*, *created an urgent need for medication*. *Antibiotics were not yet available*. *Investigations on-site showed that the indigenous people could treat dysentery successfully by oral administration of fresh and still warm camel dung*. *Eventually it was discovered that the abundant amount of B*. *subtilis led to the successful treatment*.*”*

In this internet article, the source of the camel feces story is in turn attributed to a reference we were not able to track down. Efforts to contact the author of the internet article were also unsuccessful.

Although there is considerable scientific literature on the supposed benefits of drinking camel urine [13], potential benefits of eating camel feces are not described in the scientific literature. We were able to find a single paper [14] that documents the use of camel urine, and mentions the use of camel feces.

*“The Bedouin in the Arab desert utilized to mix camel faces (sic) and urine with milk and give it to patients who were suffering from many enteric disorders and illnesses*. *They treat patients with camel faces (sic) and urine after boiling*. *Majorities of peoples (72% of peoples) drink it pure*, *whereas the remainder 28% mixes it with the milk*. *Milk was added to urine to overcome its strong odor*, *also urine must be fresh and excreted from young animals”* [14].

However, the above statement (cited verbatim) is not substantiated in the paper, the source journal is not indexed in PubMed, and the consumption of camel feces is only mentioned in the context of mixing with fresh urine.

A chapter from a book [15] that only indirectly cites the Ralph A. Lewin paper warrants an additional mention because it is used as a source in papers and introduces and expanded version of the “camel feces” story. The book chapter describes culture and drying of *Bacillus subtilis* by German soldiers to avoid the administration of fresh feces. A discussion of this book [16] by the authors mentions the “Camel feces” story and the role of *Bacillus subtilis*. This discussion contains further embellishments of the original “camel feces” story. *Bacillus subtilis* is described as a “voracious eater of other microbes”, a statement that is difficult to correlate with the limited effect a *Bacillus subtilis* probiotic has on the composition of the intestinal microbita [11]. A cross sectional analysis of 200 people from rural Thailand suggest that the presence of Bacillus subtilis, at unknown abundance, in the gastrointestinal tract reduces colonization by the pathogen *Staphylococcus aureus* [17]. However, this effect was not seen in other populations [17].

Even more puzzling is the remark that “But the *B*. *subtilis* was present only in warm dung; it would die out when the dung cooled.” Since *Bacillus subtilis* is at most a facultative anaerobe [18], it would survive after defecation and exposure of the feces to oxygen. Moreover, because *Bacillus subtilis* is a sporulating organism, its spores will certainly survive long times in fecal matter [19].

Despite the scarcity of convincing positive therapeutic clinical data with *Bacillus* probiotics and the questionable provenance of the “camel feces” story, we wanted to investigate if camel feces contains sufficient amounts of *Bacillus subtilis* vegetative cells and/or spores to be of probiotic value.

Here it should be noted that there are two main species that are designated camel, the North African camel, *Camelus dromedarius*, which has one “hump” and is also called Dromedary and the Bactrian camel, *Camelus bactrianus*, that has two “humps” and originates in central Asia. The German soldiers would have encountered Bedouins using *Camelus dromedarius’* feces.

**Fig 1:**
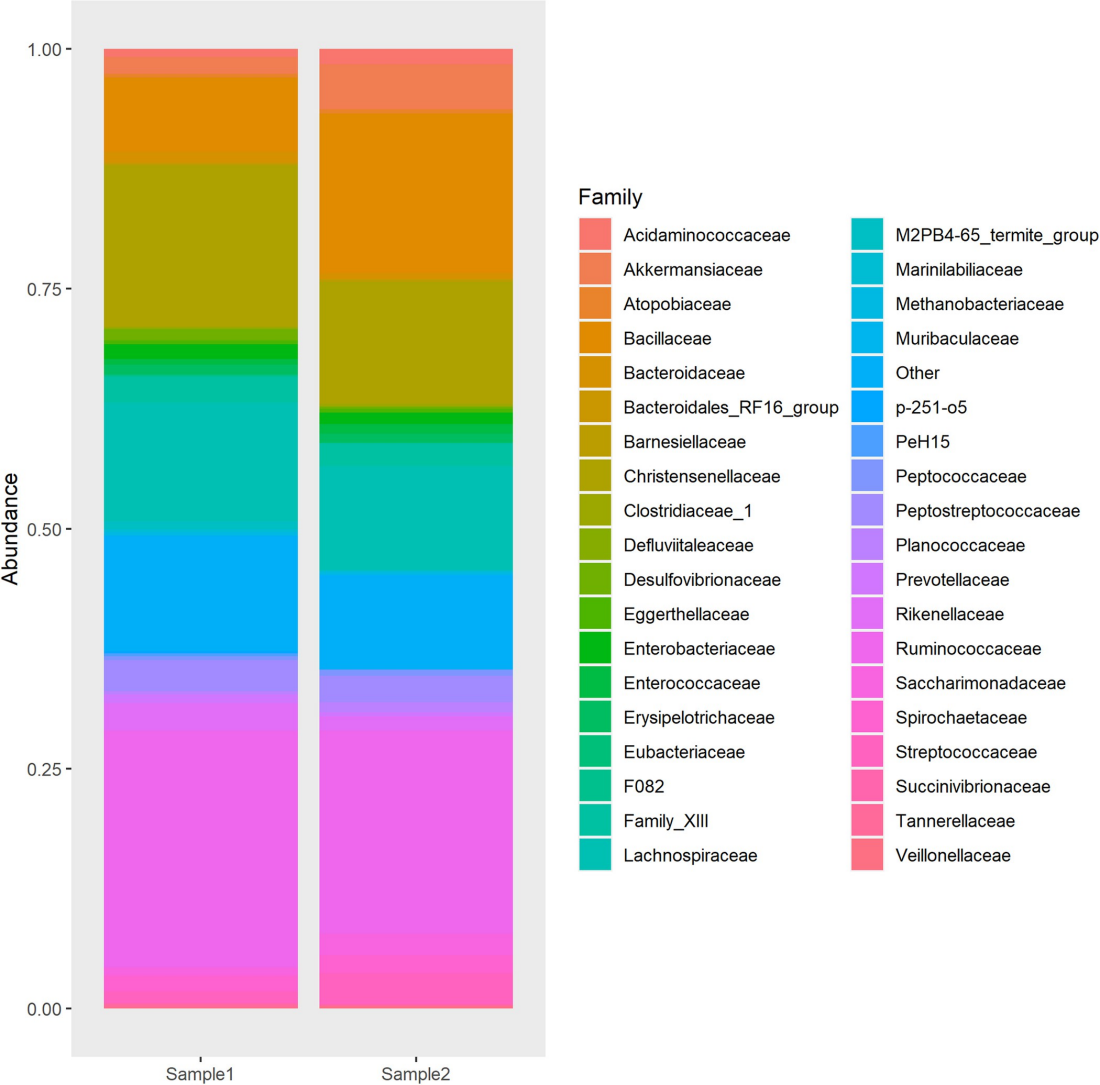
Relative abundances on family level per dromedary, determined by 16S sequencing. Only family names of the 400 most abundant amplicon sequence variants are shown, less abundant families are group under ‘Other’. The relative abundance of the Bacillus family is 0.08 and 0.16, for dromedary 1 and 2 respectively.

We therefore collected two different fecal *Camelus dromedarius* fecal samples from two different animals close to Marsa Shagra in Egypt. The samples were transported in sterile tubes and processed within 24 hours. 16S RNA analysis and MALDI-TOF were performed to determine the presence of *Bacillus subtilis*. The results of the 16S analysis are shown in Fig 1. Although there is a large abundance of *Bacillus* species, we could not detect *Bacillus subtilis*.

Our results seem to show similar results on Phylum level with the only other microbial analysis of *Camelus dromedarius* feces we were able to find [20]. Analyzing the deposited sequence data from the rectal sample described in this study using our 16S analysis pipeline failed to detect *Bacillus subtilis*.

Given the fact that the abundance of vegetative *Bacillus subtilis* might be low and could therefore be undetected due to insufficient sequence depth, we decided to determine the presence of *Bacillus subtilis* spores by plating dilutions of ethanol inactivated camel feces and identifying the colonies by MALDI-TOF.

**Fig 2:**
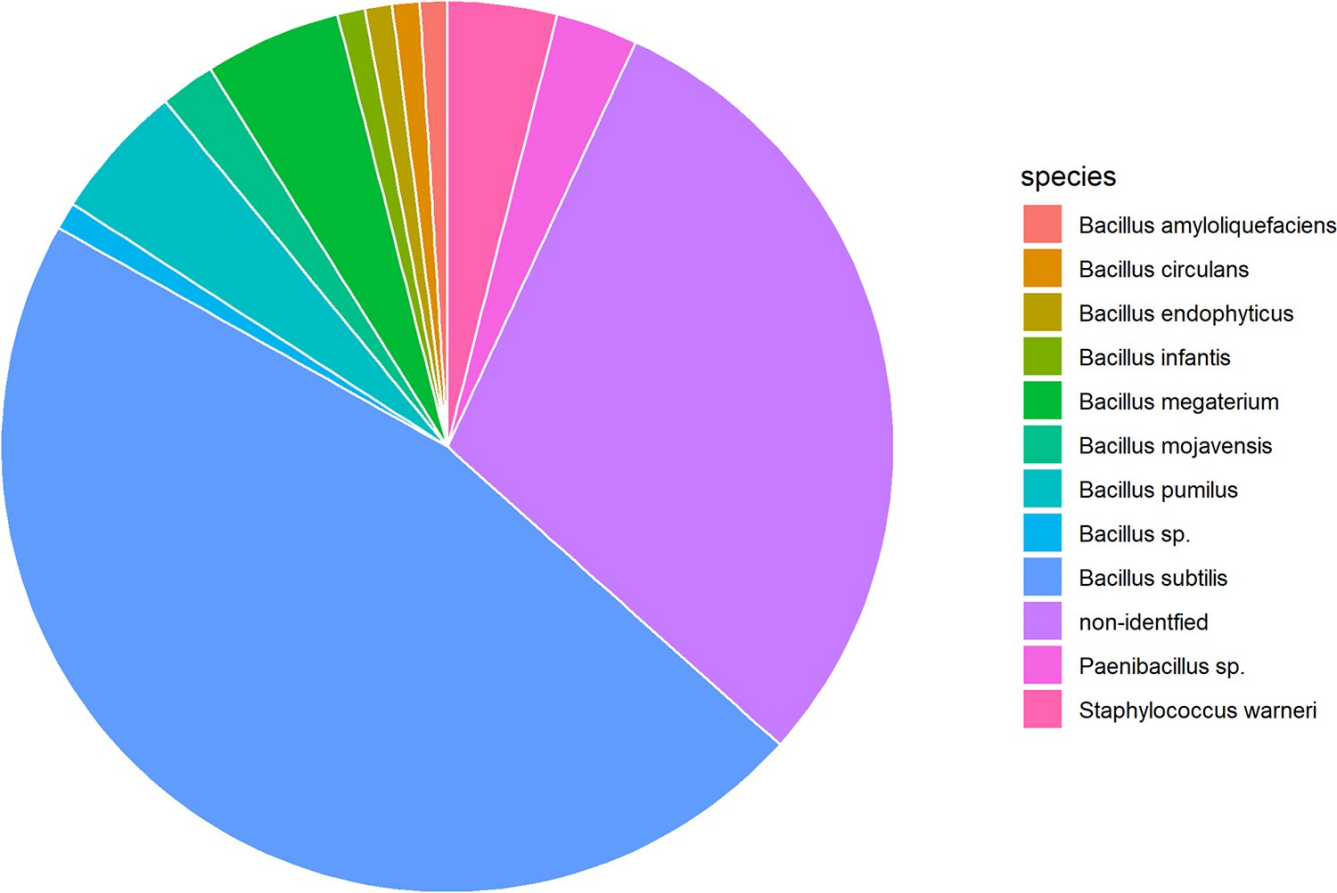
Identification of colonies growing from ethanol resistant bacterial spores by MALDI-TOF. The combined results of two different fecal samples are presented.

Indeed, using this approach we were able to detect *Bacillus subtilis*, representing 44% of all analyzed colonies, as the predominant aerobic spore former in camel feces with a concentration of spores of 1.3x10^4^ per gram feces (average of feces from 2 different camels). Fig 2. This value correlates well with the amount of *Bacillus subtilis* spores found in human feces [21]. For comparison, the average amount of ethanol resistant aerobic spore formers detected in 25 soil samples was 3.95x10^5^ per gram [22].

Based on our results and *in silico* analysis of an independent camel fecal sample we conclude that the amount of *Bacillus subtilis* in camel feces is low and comparable to that in human feces and soil.

Notably, a randomized placebo controlled trial investigated the effect of a daily dose of 2.2x10^9^ *Bacillus subtilis* spores on loose stools [11]. This study showed a modest effect on stool consistency.

Furthermore, a case report of a placebo controlled trial describes that a twice daily dose of 2x10^9^ *Bacillus subtilis* spores reduces antibiotic associated diarrhea [23].

Therapeutic effects of *Bacillus subtilis* spores were thus only seen with doses exceeding 10^9^ per day. We therefore conclude that it is unlikely that consumption of camel feces, containing 1.3x10^4^ bacillus subtilis spores per gram, would have any effect on dysentery due to its *Bacillus subtilis* content.

## Conclusions

Our research shows the emergence of a scientific “myth” that is based on a plausible but unsubstantiated reference.

We failed to find evidence that German soldiers observed the use of camel feces by Bedouins in the Second World War and have not been able to substantiate claims for a traditional use of camel feces in the treatment of dysentery.

We can rule out a role of *Bacillus subtilis* in hypothetical benefits of consuming camel feces since the amount of *Bacillus subtilis* spores in a gram of camel feces is several orders of magnitude below the therapeutical dose needed to reduce loose stools or antibiotic associated diarrhea.

Based on literature analysis and our analytical data we therefore conclude that the myth of camel feces as treatment for dysentery and its historical significance in the development of fecal transplantation are questionable and should no longer be mentioned in papers that describe the roots of fecal transplantation.

### Limitations

Our study is necessarily limited because it aims to prove a negative; Camel feces was not used in the treatment of diarrhea. Absence of evidence in our literature search does not rule out the possibility that a deeper search in historical literature will uncover evidence for the use of camel feces by Bedouins. Whereas we do show that camel feces does not contain sufficient *Bacillus subtilis* to be therapeutic, we cannot rule out the possibility that consumption of camel feces will have positive effects on dysentery due to other mechanisms.

## Methods

### Literature search

To independently investigate the use of camel feces in the treatment of diarrhea we performed an extensive literature search. Keywords used were: [camel feces diarrhea] [camel feces dysentery] [camel dung diarrhea] [camel feces dysentery] [camel feces bedouin] [camel dung bedouin]. As search platforms we used PubMed, JSTOR, Google and Google scholar.

### Fecal collection

Feces was sampled from two healthy dromedary roaming a typical desert location near Marsa Shagra in Egypt, ethical permission was therefore not needed. Samples were transported at ambient temperature in sterile tubes and processed within 24h. Approximately 200 mg feces was cut from the inside of the bolus under sterile conditions.

### DNA extraction and 16S sequencing

DNA was isolated using the PSP Spin Stool DNA Plus kit (Isogen LifeScience, The Netherlands) as follows: Approximately 100 mg feces was placed in Lysing Matrix E tubes (Bioconnect, The Netherlands) and 1.4 mL DNA stool stabilizer was added (Isogen LifeScience, the Netherlands). Samples were vortexed three repetitive rounds of 30 s at 6.5 m/s with cooling for 30s on ice in between using FastPrep bead beater (BioSPX, The Netherlands). Next, the samples were incubated for 15 min at 90 ⁰C, followed by 1 min on ice and centrifugation for 4 min at 13,000 g at room temperature. The supernatant was transferred to PSP InviAdsorb carbon filled tubes, vortexed for 15s and after 1 min on room temperature centrifuged for 6 min at 13,000 g. The supernatant was transferred to a new Eppendorf tube and centrifuged again for 6 min at 13,000 g. Then, the supernatant was transferred to a tube with 25 μl Proteinase K and incubated for 10 min at 70 ⁰C. After incubation, 400 μl binding buffer was added and first 600 μl of the sample was transferred to a column, centrifuged for 2 min at 11,000 g at room temperature. After discarding of the supernatant, the remaining sample was added on the column. The column was washed with 500 μL Wash buffer I, centrifuged for 1.5 min at 11,000 g at room temperature and the supernatant was discarded, this step was repeated with 700 μL Wash Buffer II. The column was centrifuged for another 4 min at full speed to dry the membrane and placed in a clean receiver tube. DNA was eluted in 100 μL preheated (70 ⁰C) Elution Buffer, followed by centrifugation for 2 min at 11,000 g at room temperature.

Fecal microbiota composition was profiled by sequencing the V3-V4 region of the 16S rRNA gene on an Illumina MiSeq Instrument. The data was preprocessed to ASVs with the UNOISE3 pipeline and taxonomy was added with Bayes classifier based on the Silva (v132) database [24]. Sequences classified as chloroplasts, mitochondria, contaminants and unclassified sequences were removed. Data was further analyzed by using the phyloseq [25] and ggplot2 (https://ggplot2.tidyverse.org/) packages in R 4.1.0 software (R foundation for Statistical Computing, Austria). S3 Appendix shows the annotated 16S results. The raw sequence data are deposited at the NCBI SRA: www.ncbi.nlm.nih.gov/bioproject/862696 Bioproject ID: PRJNA862696.

Illumina Miseq 16S rRNA (V3-V4 region) data from the rectum of an adult *Camelus dromedarius* from Saudi Arabia was obtained from the NCBI Sequence Read Archive, accession BioSample SAMN09784084.

Culturing and MALDI-TOF (Matrix Assisted Laser Desorption Ionization Time of Flight Mass Spectrometry) identification 1 mL 70% ethanol was added to the feces and samples were incubated for 4 hours at RT, every 45 min the samples were briefly shaken. After incubation, the samples were washed 3 times with MiliQ water and finally 5x the fecal weight in MiliQ water was added. Samples were thoroughly vortexed and filtered with a 40 μm cell strainer (Fisherbrand™, Thermo Fisher Scientific, The Netherlands). The 5x dilution was further diluted in steps of 10x till 5*10^4^. Of each dilution 20 μl was plated on LB plates *in duplo* and incubated overnight at 37 ⁰C. Colonies were counted and randomly picked for Maldi-TOF, 190 in total. The isolated colonies were deposited on a MSP 96 target plate (Bruker Daltonics Ltd, UK) with a tooth pick and pretreated with 1 μl 70% formic acid and air dried before being overlaid with matrix solution. MALDI-TOF MS was performed with a MicroFlex LT system (Bruker Daltonics Ltd, UK). Data was processed with the MALDI Biotyper MSP Identification Standard Method 1.1, the MALDI Biotyper Preprocessing Standard Method 1.1 and the Bruker MALDI-TOF Biotyper database (Bruker Daltonics Ltd, UK). Only best matches with score values ≥1.70 were included.

## Supporting information

S1 Appendix Selected papers citing the “camel feces” story. (DOCX)

S2 Appendix Original German text “Bacillus subtilis Beschreibung und Charakterisierung”. (DOCX)

S3 Appendix Annotated table of 16S sequencing results. (XLSX)

S1 Data (XLSX)
