# Supplementary material for: History of fecal transplantation; camel feces contains limited amounts of Bacillus subtilis spores and likely has no traditional role in the treatment of dysentery
Source: PLoS One. 2022 Aug 10;17(8):e0272607. doi: 10.1371/journal.pone.0272607 (PMC9365175; doi:10.1371/journal.pone.0272607)
Supplement: S1 Appendix — (DOCX) [file pone.0272607.s001.docx]

**Appendix 1**, selected papers citing the “camel feces” story.

1. Evrensel, A. & Ceylan, M. E. Fecal microbiota transplantation and its usage in neuropsychiatric disorders. *Clinical Psychopharmacology and Neuroscience* vol. 14 231–237 (2016).

2. Baktash, A. *et al.* Mechanistic insights in the success of fecal microbiota transplants for the treatment of Clostridium difficile infections. *Frontiers in Microbiology* vol. 9 1242 (2018).

3. Damman, C. J., Miller, S. I., Surawicz, C. M. & Zisman, T. L. The microbiome and inflammatory bowel disease: Is there a therapeutic role for fecal microbiota transplantation. *Am. J. Gastroenterol.* **107**, 1452–1459 (2012).

4. de Groot, P. F., Frissen, M. N., de Clercq, N. C. & Nieuwdorp, M. Fecal microbiota transplantation in metabolic syndrome: History, present and future. *Gut Microbes* vol. 8 253–267 (2017).

5. D’Odorico, I. *et al.* Role of fecal microbiota transplantation in inflammatory bowel disease. *Journal of Digestive Diseases* vol. 19 322–334 (2018).

6. Smits, L. P., Bouter, K. E. C., De Vos, W. M., Borody, T. J. & Nieuwdorp, M. Therapeutic potential of fecal microbiota transplantation. *Gastroenterology* vol. 145 946–953 (2013).

7. D. Goldenberg, S. & Merrick, B. The role of faecal microbiota transplantation: looking beyond Clostridioides difficile infection. *Therapeutic Advances in Infectious Disease* vol. 8 (2021).

8. Wang, A. Y., Popov, J. & Pai, N. Fecal microbial transplant for the treatment of pediatric inflammatory bowel disease. *World Journal of Gastroenterology* vol. 22 10304–10315 (2016).

9. Lyon, L. ‘All disease begins in the gut’: Was Hippocrates right? *Brain* **141**, e20 (2018).

10. Sbahi, H. & Di Palma, J. A. Faecal microbiota transplantation: applications and limitations in treating gastrointestinal disorders. *BMJ Open Gastroenterol.* **3**, e000087 (2016).

11. Ettinger, G., Burton, J. P. & Reid, G. If microbial ecosystem therapy can change your life, what’s the problem? *BioEssays* **35**, 508–512 (2013).

12. Gasbarrini, G., Bonvicini, F. & Gramenzi, A. Probiotics History. *J. Clin. Gastroenterol.* **50**, S116–S119 (2016).

13. Helmink, B. A., Khan, M. A. W., Hermann, A., Gopalakrishnan, V. & Wargo, J. A. The microbiome, cancer, and cancer therapy. *Nature Medicine* vol. 25 377–388 (2019).

14. Keskey, R., Cone, J. T., DeFazio, J. R. & Alverdy, J. C. The use of fecal microbiota transplant in sepsis. *Translational Research* vol. 226 12–25 (2020).

15. Bibbò, S. *et al.* Fecal Microbiota Transplantation: Screening and Selection to Choose the Optimal Donor. *J. Clin. Med.* **9**, 1757 (2020).

16. Keskey, R., Cone, J. T., DeFazio, J. R. & Alverdy, J. C. The use of fecal microbiota transplant in sepsis. *Translational Research* vol. 226 12–25 (2020).

17. Gulati, M., Singh, S. K., Corrie, L., Kaur, I. P. & Chandwani, L. Delivery routes for faecal microbiota transplants: Available, anticipated and aspired. *Pharmacological Research* vol. 159 104954 (2020).

18. Stripling, J. & Rodriguez, M. Current Evidence in Delivery and Therapeutic Uses of Fecal Microbiota Transplantation in Human Diseases—Clostridium difficile Disease and Beyond. *Am. J. Med. Sci.* **356**, 424–432 (2018).

19. Hanssen, N. M. J., de Vos, W. M. & Nieuwdorp, M. Fecal microbiota transplantation in human metabolic diseases: From a murky past to a bright future? *Cell Metabolism* vol. 33 1098–1110 (2021).

20. Ser, H. L., Letchumanan, V., Goh, B. H., Wong, S. H. & Lee, L. H. The Use of Fecal Microbiome Transplant in Treating Human Diseases: Too Early for Poop? *Frontiers in Microbiology* vol. 12 1005 (2021).

21. D. Goldenberg, S. & Merrick, B. The role of faecal microbiota transplantation: looking beyond Clostridioides difficile infection. *Therapeutic Advances in Infectious Disease* vol. 8 (2021).

22. S, C. Microbial Treatments for the Mind. *IEEE Pulse* **10**, 3–7 (2019).

23. Khajah, M. A. The potential role of fecal microbiota transplantation in the treatment of inflammatory Bowel disease. *http://dx.doi.org/10.1080/00365521.2017.1347812* **52**, 1172–1184 (2017).

24. Evrensel, A. & Ceylan, M. E. Fecal Microbiota Transplantation in the Treatment-Resistant Psychiatric Disorders. *Treat. Resist. Psychiatry Risk Factors, Biol. Manag.* 369–376 (2019) doi:10.1007/978-981-10-4358-1_24.

25. Gagliardi, A. *et al.* Rebuilding the gut microbiota ecosystem. *International Journal of Environmental Research and Public Health* vol. 15 1679 (2018).

26. Ettinger, G., Burton, J. P. & Reid, G. If microbial ecosystem therapy can change your life, what’s the problem? *BioEssays* **35**, 508–512 (2013).

27. Vandenplas, Y., Pierard, D. & De Greef, E. Fecal Microbiota Transplantation: Just a Fancy Trend? *J. Pediatr. Gastroenterol. Nutr.* **61**, 4–7 (2015).

28. Scott, K. P., Antoine, J.-M., Midtvedt, T. & van Hemert, S. Manipulating the gut microbiota to maintain health and treat disease. *Microb. Ecol. Heal. Dis.* **26**, (2015).

29. Borgia, G., Maraolo, A. E., Foggia, M., Buonomo, A. R. & Gentile, I. Fecal microbiota transplantation for Clostridium difficile infection: Back to the future. *Expert Opinion on Biological Therapy* vol. 15 1001–1014 (2015).

30. Vaishnavi, C. Fecal microbiota transplantation for management of Clostridium difficile infection. *Indian Journal of Gastroenterology* vol. 33 301–307 (2014).
