# Supplementary material for: History of fecal transplantation; camel feces contains limited amounts of Bacillus subtilis spores and likely has no traditional role in the treatment of dysentery
Source: PLoS One. 2022 Aug 10;17(8):e0272607. doi: 10.1371/journal.pone.0272607 (PMC9365175; doi:10.1371/journal.pone.0272607)
Supplement: S2 Appendix — (DOCX) [file pone.0272607.s002.docx]

**Appendix 2**, original German text “Bacillus subtilis Beschreibung und Charakterisierung”

*“Die erste dokumentierte medizinische Anwendung von B. subtilis erfolgte 1941 durch die Sanitätsabteilung des Afrikakorps der deutschen Wehrmacht während des Feldzuges in Libyen. Weil einer Ruhr-Epidemie zahlreiche Soldaten zum Opfer fielen, bestand der dringende Bedarf an Medikamenten. Antibiotika waren noch nicht verfügbar. Nachforschungen vor Ort ergaben, daß die Ruhr bei der einheimischen Bevölkerung erfolgreich durch die orale Verabreichung frischen, noch warmen Kameldungs behandelt wurde. Für den Behandlungserfolg machte man den schließlich in großer Zahl gefundenen B. subtilis verantwortlich.”*
